# Recovery of Bioactive Ellagitannins by Ultrasound/Microwave-Assisted Extraction from Mexican Rambutan Peel (*Nephelium lappaceum* L.)

**DOI:** 10.3390/molecules27051592

**Published:** 2022-02-28

**Authors:** Luis Estrada-Gil, Juan C. Contreras-Esquivel, Carolina Flores-Gallegos, Alejandro Zugasti-Cruz, Mayela Govea-Salas, Marco A. Mata-Gómez, Raúl Rodríguez-Herrera, Juan A. Ascacio-Valdés

**Affiliations:** 1 Food Reasearch Department, School of Chemistry, Autonomous University of Coahuila, Ing. J. Cárdenas Valdéz S/N, República, Saltillo 25280, Mexico; estrada.l@uadec.edu.mx (L.E.-G.); carlos.contreras@uadec.edu.mx (J.C.C.-E.); carolinaflores@uadec.edu.mx (C.F.-G.); alejandro_zugasti@uadec.edu.mx (A.Z.-C.); raul.rodriguez@uadec.edu.mx (R.R.-H.); 2 Laboratory of Nanobiociences, School of Chemistry, Autonomous University of Coahuila, Ing. J. Cárdenas Valdéz S/N, República, Saltillo 26280, Mexico; m.govea.salas@uadec.edu.mx; 3 School of Engineering and Science, Instituto Tecnológico y de Estudios Superiores de Monterrey, Campus Puebla, Atlixcáyotl 5718, Reserva Territorial Atlixcáyotl, Puebla 72453, Mexico; mmatag@tec.mx

**Keywords:** rambutan peel, antioxidant, geraniin, emerging technologies

## Abstract

Rambutan (*Nephelium lappaceum* L.) is a tropical fruit from Asia which has become the main target of many studies involving polyphenolic analysis. Mexico produces over 8 million tons per year of rambutan, generating a huge amount of agro-industrial waste since only the pulp is used and the peel, which comprises around 45% of the fruit’s weight, is left behind. This waste can later be used in the recovery of polyphenolic fractions. In this work, emerging technologies such as microwave, ultrasound, and the hybridization of both were tested in the extraction of phenolic compounds from Mexican rambutan peel. The results show that the hybrid technology extraction yielded the highest polyphenolic content (176.38 mg GAE/g of dry rambutan peel). The HPLC/MS/ESI analysis revealed three majoritarian compounds: geraniin, corilagin, and ellagic acid. These compounds explain the excellent results for the biological assays, namely antioxidant activity evaluated by the DPPH, ABTS, and LOI (Lipid oxidation inhibition) assays that exhibited great antioxidant capacity with IC_50_ values of 0.098, 0.335, and 0.034 mg/mL respectively, as well as prebiotic activity demonstrated by a *µMax* (maximum growth) of 0.203 for *Lactobacillus paracasei*. Lastly, these compounds have shown no hemolytic activity, opening the door for the elaboration of different products in the food, cosmetic, and pharmaceutical industries.

## 1. Introduction

Rambutan (*Nephelium lappaceum* L.) is a tropical fruit from Southeast Asia, specifically from Malaysia. Because of its tasty flavor, rambutan was introduced to other parts of Asia, Africa, Central America, and Mexico. In Mexico, it grows from seeds in Soconusco, Chiapas, and has commercial applications in the states of Chiapas, Tabasco, Guerrero, Colima, San Luis Potosi, Nayarit, and Michoacán [1]. The plantation and growth of rambutan is a tremendous economic activity in Southern Mexico, where it reached production levels of 9800 tons in 2018. The recollected fruits are exported to the United States of America (USA), Japan, Belize, Germany, the United Kingdom, Canada, and El Salvador. In the same year, exportations of Mexican rambutan to the U.S.A. reached 4491 tons (815 USD/ton), generating an estimated revenue of 3.7 million dollars [2].

On average, rambutan fruit weight is between 27 and 30 g. The main constituents of rambutan are the peel, pulp, seed, and embryo, in which the peel and the pulp represent around 45.7% and 44.8% of the fruit’s total weight, respectively [3]. It is noteworthy to mention that many commercial products derived from rambutan are produced all around the world. For example, jams, liquors, preserved fruits in syrup, and jellies can be made from rambutan pulp [4]. However, the production of these goods generates a vast amount of agro-industrial waste by leaving behind the peel, seed, and embryo, which could be used in other applications [5].

In some fruits, the peel has higher bioactivity than the edible portion of the fruit, and rambutan’s peel is not an exception. Studies conducted from biological assays have proved the great potential of this agricultural waste as an antioxidant, antimicrobial, antiviral, anticancer, anti-allergic, anti-obesity, antidiabetic, anti-arthritic, as well as a promoter of cardiovascular health [6,7,8,9,10,11,12]. Despite the proven properties of rambutan, its waste has been little exploited. Previous studies have identified that the main reason behind these beneficial biological activities is the high amount of polyphenolic content in rambutan’s peel, such as geraniin, as the majoritarian compound, ellagic acid, and corilagin [13]. Ellagitannins are part of traditional medicine all around the world; they are present in plants with a high content of geraniin. In Japan, people use *Geranium thunbergii*, a geraniin-rich plant, as an antidiarrheal drug [14], while in Mexico, “Pata de Leon”, a geraniin-rich plant, is also used to treat fever, pain, and gastrointestinal disorders [15]. In India, *Phyllanthus emblica* is also used to prevent peptic ulcers and alopecia [16]. Lastly, in Malaysia, rambutan fruit and shell are used to treat dysentery and diarrhea, as well as astringent, stomachic, and antihelmintic agents [17].

Conventional extraction technologies by using solvents are an essential step in the recovery of high added value compounds from plant materials. However, conventional extraction methods, such as maceration and Soxhlet, are time-consuming approaches that have low efficiency and use an incredible amount of solvent. Nowadays, the extraction of bioactive compounds can be done with new and emerging technologies, such as microwave-assisted extraction (MAE) and ultrasound-assisted extraction (UAE). These techniques reduce extraction times and lead to better yields with lower use of solvents, resulting in low energy consumption, thus making these technologies less expensive and environmentally friendly [18,19]. UAE is based on the formation and subsequent breakage of bubbles caused by cavitation—a phenomenon created by the propagation of acoustic waves at high frequencies (>20 kHz). The implosion of bubbles results in mechanical, chemical, and physical effects on the biological material, releasing the bioactive compounds. On the other hand, MAE bursts the cellular structure thanks to the heat irradiation produced by the vibration of the water molecules in the medium, thus releasing the compounds on the medium. Although this method has proven to be effective [20,21], high temperatures can cause degradation of polyphenol content when exposed to long periods. A combination of previous technologies creates a “hybrid” extraction technology that takes advantage of both technologies and, at the same time, creates a seamless interaction between the cavitation bubbles formed by the UAE and the high temperatures granted by the MAE, thus presenting even better results than when the technologies are used separately [22]. These emerging technologies, such as microwave, ultrasound, and the hybridization of both, represent a major advance to obtain compounds of interest from plant materials and encompass more efficient, greener, and cheaper options than conventional technologies because better yields can be obtained from them with even fewer resources.

Hybrid extraction technologies have been used previously in the extraction of phenolic compounds from fruit peels such as mango. In a study, carried out by Ordoñez-Torres et al., 54.15 mg/g of total polyphenolic content was obtained [23]. Another study established that Mexican rambutan peel extraction by hybrid technology [24] obtained a total of 334.1 mg/g of polyphenolic content, suggesting a great potential in future applications.

Regarding the hybrid extraction technology of MAE and UAE, future applications have no limit because this is an environmentally friendly technology, as it can work with solvents as noble as water to avoid using more contaminating substances such as hexane. Using hybrid technology helps to increase solubility and obtain better extracting yields in less time than conventional technologies, such as maceration or Soxhlet. Obtaining better yields facilitates the production of better products. Hybrid technology opens the way to create better products thanks to the far better yielding that it grants. This technology could be used in future applications in the food, pharmaceutical and cosmetology industries as they benefit from the creation of products with high antioxidant content [25]. This study aims to obtain, recover, and identify the high added value of biological compounds contained within the rambutan peel by using microwave, ultrasound, and hybrid emergent technologies, as well as to compare the efficiency of these three methods and characterize the biological properties of these compounds in terms of antioxidant, prebiotic, and anti-hemolytic activities for further applications in cosmetic, food and pharmaceutical industries.

## 2. Results and Discussion

### 2.1. Polyphenolic Compounds Determination

**Figure 1:**
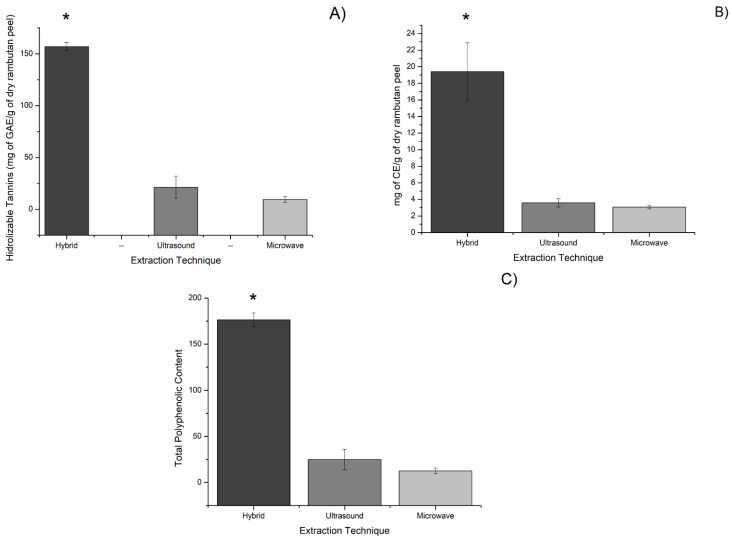
Extraction of polyphenolic compounds from Mexican rambutan peel. Figure (**A**) represents hidrolyzable tannins as mg of gallic acid equivalents/g of dry rambutan peel, Figure (**B**) represents condensed tannins as mg of catechin equivalents/g of dry rambutan peel, and Figure (**C**) represents the total amount of polyphenolic content found in the extraction technique, (*) indicates significant differences (*p* < 0.05) between extraction techniques.

Three extraction methods, UAE, MAE, and hybrid (ultrasound and microwave combined) assisted extractions, were used and compared in terms of phenol extraction efficiency (Figure 1).

Data presented in Figure 1 is expressed as the mean of three replications and ±standard deviation. A mean comparison test was carried out for all extraction methods analyzed and the results show that there is a significative difference between hybrid technology and MAE and UAE in every assay. This determined that the combination of UAE and MAE was the best yielding technology by far, as the hybrid technology was significatively different from the other two technologies, while no significative differences could be found between the results of UAE and MAE.

It was observed that the combination of UAE and MAE technologies showed great potential to extract polyphenols. The results are represented as mg of polyphenolic content for every g of dry rambutan peel. For the hydrolyzable polyphenolic content, hybrid extraction yielded an average of 156.96 mg of GAE/g of dry rambutan peel, followed by UAE with an average of 21.32 mg of GAE/g of dry rambutan peel. Lastly, the MAE technology yielded 9.48 mg of GAE/g dry rambutan peel. In the case of condensed polyphenolic content, hybrid extraction was again the best among the three extraction techniques, obtaining an average of 19.41 mg of CE/g of dry rambutan peel, followed by UAE with an average of 3.58 mg of CE/g of dry rambutan peel, and MAE with 3.07 mg of CE/g of dry rambutan peel. Analyzing the hydrolyzable and condensed polyphenolic content of the three extracts, it was determined that hybrid extraction technology was the best all-around, obtaining 176.38 mg/g, followed by UAE with 24.90 mg/g, and lastly, MAE with 12.56 mg/g (Figure 1). The majority of the polyphenols contained within the extract were hydrolyzable polyphenols with 88.98% of the total, compared to studies, done by Hernandez et al., to Mexican rambutan peel [26], where the sum of hydrolyzable polyphenols represented 78% of the total content.

The polyphenolic content found in the hybrid extraction of the Mexican rambutan peel proved to be greater than that found in other tropical fruits, such as mango Ataulfo peel, where 35.93 mg/g of hydrolyzable and 18.21 mg/g of condensed polyphenols were found. In a study, by Ordoñez-Torres et al. [23], extractions were also performed by hybrid extraction technology, resulting in an improved extraction yield compared to results obtained by conventional technologies.

A study, carried out by Phuong et al. [27], found that UAE was effective in the extraction of polyphenolic compounds from rambutan peel of the Java variety (Can Tho City, Vietnam), achieving a total polyphenolic content of 0.14–0.16 mg per g of rambutan peel. By using the same technology, the Mexican variety yielded 0.17 mg per g of rambutan peel. Therefore, a combination of ultrasound and microwave technologies resulted in a higher extraction yield of polyphenols from the Mexican variety, as demonstrated in this work. The following experiments were carried out using the highest yielding extraction, which was the one obtained by the hybridization of ultrasound and microwave technologies. 

### 2.2. Identification of Rambutan Peel Polyphenols

To identify the compounds present in the extract obtained with the combination of ultrasound and microwave technologies, and fractionated using Amberlite XAD-16 chromatography, an HPLC/ESI/MS (High Performance Liquid Cromatography/Electrospray Ionization/Mass Spectrometry) analysis was performed. The HPLC/ESI/MS analysis allowed five compounds, four ellagitannins, and one hydroxybenzoic acid dimer to be identified (Table 1). 

**Figure 2:**
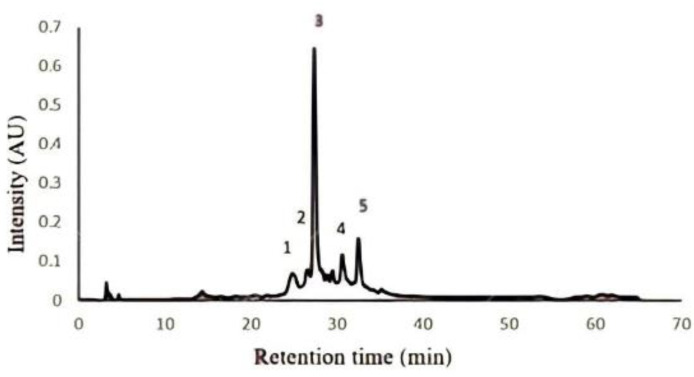
Chromatogram obtained from the HPLC/ESI analysis of the rambutan peel extract. Each number represents a component found in the chromatogram as follows: (1) dimmers of tergallagic-*O*-hexoside, (2) corilagin, (3) geraniin, (4) ellagic acid pentoside, and (5) ellagic acid.

Of the five polyphenolic compounds identified in our work, the three main compounds were found to be geraniin as the majoritarian compound, followed by corilagin, and ellagic acid. Figure 2 shows the results of the chromatogram with the peaks of the identified compounds. 

The majoritarian compounds (geraniin, corilagin, and ellagic acid) have been previously identified on rambutan peel and studied for their wide range of biological activities. In a previous study, Hernandez-Hernandez et al. [24], were able to recover seven polyphenolic compounds using Mexican rambutan husk, obtaining corilagin, geraniin, ellagic acid, ellagic acid pentoside, punigluconin, tetragalloyglucose, and pedunculagin. In another study, Mendez-Flores et al. [19], recovered 12 polyphenolic compounds, namely gallic acid, breviform carboxylic acid, ellagic acid, gallic acid 3-*O*-gallate, isorhamnetin 3-*O*-glucoside 7-*O*-rhamnoside, galloyl-HHDP-hexoside, corilagin, pedunculagin, ellagic acid derivate, theaflavin 3,3′-*O*-digallate, galloyl-bis-HHDP-hexoside (casuarinin), and geraniin, using UAE to extract polyphenols from Mexican rambutan husk. It is important to mention that in these two studies, the three majoritarian compounds were also geraniin, corilagin, and ellagic acid, which are also the three majoritarian compounds in this study.

In another study, Palanisamy et al. [12], described the compounds found within the Malaysian variety of the rambutan husk, finding only three compounds, geraniin, corilagin, and ellagic acid, while Lestari et al. [8], analyzed the polyphenolic compounds from the Java rambutan variety, also revealing the presence of the previous three compounds. These studies prove that the husk of the Mexican rambutan variety possesses more polyphenolic compounds than other Asian varieties that may provide potential biological activities such as antioxidant, antimicrobial, antiviral, anticancer, anti-allergic, anti-obesity, antidiabetic, anti-arthritic, and improvement on cardiovascular health [6,28,29,30,31,32,33,34,35].

### 2.3. Antioxidant Assays

Antioxidant assays were only performed using the polyphenolic content gathered from the Mexican rambutan peel subjected to hybrid extraction. ABTS and DPPH scavenging activity, as well as lipid oxidation inhibition (LOI), were used to study the antioxidant capacity of the rambutan peel extract. Here, the antioxidant activity was determined by the half-maximal inhibitory concentration (IC_50_) of ABTS, DPPH, and LOI. Table 2 summarizes the antioxidant capacity of the Mexican rambutan peel extract.

Data were gathered from the mean of three replicates and the standard deviation. The Tukey mean comparison test results showed that there is a significant difference between our reference antioxidant Trolox and Mexican rambutan peel extract. The extract had better antioxidant activity in the ABTS and DPPH assays and no significant difference was found in the LOI assay.

The ABTS and DPPH scavenging activity obtained was higher than that reported by Ling et al. [36], where an IC_50_ of 3.04 ± 0.03 mg/mL was obtained for the DPPH free radical scavenging activity and 8.3 ± 0.07 mg/mL for the ABTS free radical scavenging activity; this can be explained because the rambutan evaluated by these authors was a different species. The Mexican rambutan grows under foreign soil, climate, and humidity conditions that differ from the Malaysian variety. Regarding the maximum inhibition percentage obtained for the LOI assay, a significant 89% of inhibition was achieved. This is comparable to the study of Mendez-Flores et al. [19], where Mexican rambutan peel showed a maximum inhibition percentage of 91.74%. These results can be explained because the rambutans peel used for the assay was from the same Mexican variety. This study confirms the fact that polyphenolic extracts from the Mexican rambutan peel have an excellent lipid oxidation inhibition activity, which can prevent human body diseases caused by cellular oxidation processes.

Mexican rambutan peel polyphenolic extract possesses a tremendous antioxidant potential exhibited by the previously obtained results, which can be attributed to the previously identified geraniin, corilagin, and ellagic acid. Antioxidant activity not only refers to free radical scavenging, but also inhibits lipid oxidation, which can cause the degradation of cellular walls. Based on the results, rambutan peel extract can compete in antioxidant capacity with Trolox as 2 out of 3 assays showed that the polyphenolic extract had better results than a known antioxidant of reference such as Trolox.

### 2.4. Prebiotic Assays

The prebiotic assays showed different results for both bacteria analyzed, *Lactobacillus brevis* and *Lactobacillus paracasei* (Table 3). 

For *L*. *paracasei*, there was no significant difference between *µMax* of the positive control and the 1000 and 500 ppm treatments. Additionally, the 125 and 250 ppm treatments showed lower growth compared to that of the 500 and 1000 ppm treatments, but it is possible that these concentrations show a prebiotic effect. Meanwhile in *L*. *brevis*, none of the treatments analyzed in the assay proved to have a prebiotic effect, and actually prevented its growth, showing an antibiotic effect on this bacterial strain.

The best effect of Mexican rambutan peel polyphenolic extract on bacterial growth was for *L*. *paracasei*, obtaining a *µMax* of 0.203 very similar to that of the positive control, which was 0.204. Some of the concentrations did not cause the probiotic bacteria *L. paracasei* to grow as expected and only some of the concentrations, i.e., 125, 250, 500, and 1000 ppm, were found to be benefical for the prebiotic effect. The best result was achieved when 500 and 1000 ppm of rambutan peel extract was used according to the Tukey mean comparison test where the 125, 250, 500, and 1000 ppm treatments were no different from the positive control, meaning that the polyphenolic content could have prebiotic activity with *L*. *paracasei* probiotic bacteria at certain concentrations when properly administered. On the other hand, *L. brevis* was not possitively affected by most of the concentrations and did not have an expected growth. Out of all the treatments, only the 31 ppm treatment stands above all, with a *µMax* of 0.19, and all the other concentrations do not favor the probiotic bacterial growth. With these results, it can be implied that rambutan peel extract can selectively benefit the proliferation of certain probiotic bacteria that can be useful in the human gastrointestinal tract. A study, carried out by Parkar et al. [37], shows that polyphenolic compounds affect some bacteria such as *Lactobacillus rhamnose* and pathogenic bacteria such as *Staphylococcus aureus* and *Escherichia coli*, where the polyphenolic compounds showed more inhibition activity over the pathogenic bacteria. They did not affect the growth of the *L. rhamnosus* strain, proving that polyphenolic compounds may have a possible prebiotic effect. Another study, carried out by Jiao et al. [38], determined that blueberry polyphenolic extracts can be potentially used as a prebiotic, modulating the intestinal microbiota, specifically regulating the growth of *Bifidobacterium* and other pathogenic bacteria such as *Desulfovibrio*, *Adlercreutzia*, *Helicobacter*, *Flexispira*, and *Prevotella*.

Some bacteria which are isolated from the human gut microbiome possess enzymes that can metabolize polyphenolic content as a carbon source and may stimulate the efficient use of alternative methods i.e., a-ramnosidase, b-galactosidase, and b-glucuronidase [39]. Particularly, certain species of the *Lactobacillus* genre possess a gallate decarboxylase that degrades gallic acid in pyrogallol [40], which degrades and can enter the Krebs cycle. Galic acid also degrades to oxalacetic acid and piruvate [41]. Other enzymes, such as esterases, hydrogenases, dehydrogenases, dehydroxylases, decarboxylases, and isomerases, are responsible for decomposing the structures of polyphenolic content into carbon C3 intermediates [42].

Based on the results obtained by the prebiotic assay, Mexican rambutan peel extract shows prebiotic activity selectively only at certain concentrations, whereas some bacteria such as *L*. *brevis* show an antibiotic effect when in contact with the polyphenolic extract, and bacteria such as *L*. *paracasei* show growth at high polyphenolic concentrations such as 500 and 1000 ppm of Mexican rambutan peel extract.

### 2.5. Hemolytic Activity

Plants have been reported to be an excellent source of phenolic compounds, which are deeply related to antioxidant activities, but they are useless if the extracts present any side effects such as hemolysis, an indicator of erythrocytes lysis [43]. Hemolysis assays showed that Mexican rambutan husk polyphenolic extract and its compounds are not hemolytic and pose no threat to human blood cells if consumed. All the analyzed concentrations showed 0% of hemolytic activity. To compare the information, a negative and positive control was used. Table 4 exhibits the results for the hemolytic activity. 

Data in Table 4 is expressed as the mean of three replicates ± standard deviation. A Tukey means comparison test with a significance level of *p* < 0.05 was realized and none of the results shown for the rambutan peel extract were found to be significantly different from one another.

These results can be compared to a study, conducted by Krishna et al. [44], where a Mulberry fruit polyphenolic extract exhibited a hemolysis percentage of 53.98 ± 0.06 when 100 µg/mL were used. Another study, conducted by Halla et al. [45], showed that the essential oils of some medicinal plants from Algeria, such as *Pituranthos scoparius*, *Myrtus nivelllei*, *Rosmarinus officinalis*, and *Mentha piperita*, show 93.00, 72.88, 72.75, and 43.26% of hemolytic activity, respectively, when using a concentration of 3.12 mg/mL. This was achieved after incubating the extract with the red cells for 120 min at 37 °C. The fact that the Mexican rambutan peel extracts exhibited no hemolysis is a promising result that can be related to the high amount of hydrolyzable polyphenols with a high antioxidant activity, which allows them to have protective effects against oxidative damage and keep the membrane of red blood cells intact [46].

## 3. Materials and Methods

### 3.1. Preparation of Material and Extraction of Polyphenols

The material used was Mexican rambutan (*Nephelium lappaceum* L.) peel gathered directly from the fruit, which was obtained from the region of Soconusco, Chiapas, Mexico. Once received, the peels were dried up in a conventional oven at 50 °C for 48 h, and after that, the dried shells were grounded into a fine powder, which was then homogenized at a fine particle size of 2 mm.

The extracts were obtained using three emergent technologies: MAE, UAE, and the combination of the previous technologies, to maintain a greener and cleaner aspect in the project. The conditions used for the extraction with the three technologies were previously defined in the workgroup by Hernandez-Hernandez et al. [24]. Briefly, in all cases, water was used as a solvent with a 1:16 mass/volume ratio (one liter of water and 62.5 g of rambutan peel powder). For UAE, the equipment was operated at a frequency of 25 kHz for 20 min. In the case of MAE, the machine was operated at 2450 MHz and extraction time was 5 min until it reached a target temperature of 70 °C, thus maintaining the polyphenolic compounds intact as they were unaffected due to the low exposure time to the high temperature; prolonged periods of high temperatures can lead to degradation and loss of yield and quality [47]. Extractions of polyphenolic content by combining ultrasound and microwave-assisted extraction were performed with a hybrid technology system, that is, the Ultrasound/Microwave Cooperative Workstation (Nanjing ATPIO Instruments Manufacture Co., Ltd. Company, Nanjing, China). For the hybrid-assisted extraction, the same conditions used for the ultrasound and microwave-assisted extractions were employed simultaneously.

After extraction, all the extracts were filtered in a dark room to protect the phenolic compounds and remove any remains of rambutan peel which could alter the final results. Once they were filtered, the extracts were placed in a glass bottle covered with aluminum for further protection from light and stored at −18 °C.

### 3.2. Analytical Procedures

The best extraction technology was determined by measuring the total soluble and bound polyphenolic content, which was obtained by summing the hydrolyzable and condensed polyphenols. 

The hydrolyzable polyphenols were measured by the Folin-Ciocalteu method [48]. Briefly, 400 μL of Folin-Ciocalteu reagent were added to 400 μL of the sample. After 5 min, 400 μL of Na_2_CO_3_ were added to the mixture. After 1 min, 2.5 mL of distilled water was added. The absorbance of the samples was measured at 790 nm by using a 1 cm cell in a Biomate 3 spectrophotometer (Thermo Spectronic). This process was carried out by triplicate, and the results were expressed as the mean mg of gallic acid equivalents per g of dry rambutan peel. Gallic acid was used as a standard in a range of 0–1000 ppm. The condensed polyphenols were assayed by the HCl butanol method, according to Shay et al. [49]. Sample volumes of 500 µL were transferred into screw cap test tubes of maximum 10 mL capacity. Then, 3 mL of HCl-Butanol 1:10 solution were added to the mixture. Subsequently, 100 μL of a 1:9 ferric solution were added. Then, the tubes were mixed and placed in a water bath at 100 °C for 1 h. Next, the solutions were cooled down at room temperature, and finally, the absorbance of the samples and catechin standards (0–1000 ppm) were read at 460 nm. The process was carried out in triplicate.

### 3.3. Chromatography Fractionation

Hybrid technology yielded the highest extraction of polyphenols. Therefore, the extract obtained by using this technology was subjected to chromatography fractionation in a glass column (60 cm height and 2.5 cm diameter) packed with Amberlite XAD-16 to recover the compounds of interest. The procedure was performed according to the methodology reported by Ascacio-Valdes et al. [50]. First, the column was placed in a stand and kept vertically. The column was filled with the mobile phase. Then, a sample load of 800 mL was poured into the column and the excess of solvent was let to fluid out of the column by gravity. Subsequently, a washing step with distilled water was done to remove the undesirable compounds, which were unretained in the amberlite sorbent. To obtain the compounds of interest, an elution with 96% (*v*/*v*) ethanol was carried out to recover the polyphenol-rich fraction in a flask. Then, ethanol was evaporated at 50 °C for 24 h in a traditional oven to recover the polyphenolic fraction as a powder. 

### 3.4. HPLC/MS Identification

Identification of the polyphenolic compounds obtained from the rambutan peel extract was carried out with the method described by Hernandez-Hernandez et al. [24]. Mexican rambutan extract powder (300 mg) containing polyphenols was prepared in a 2 mL solution of 50% ethanol. Then, the volume was adjusted to 10 mL with distilled water and the solution was filtered using 0.45 μm nylon membranes. The samples were analyzed by reversed-phase high-performance liquid chromatography in a Varian HPLC system, including an auto-sampler (ProStar 410, Varian, Palo Alto, CA, USA), a ternary pump (ProStar 230I, Varian, Palo Alto, CA, USA), and a PDA (Photo Diode Array) detector (ProStar 330, Varian, Atlanta, GA, USA). A chromatography ion trap mass spectrometer (Varian 500-MS I.T. Mass Spectrometer, Palo Alto, CA, USA) equipped with an electrospray ion source, coupled with the HPLC system, was also used. The samples (5 μL) were injected onto a preequilibrated Denali C18 column (150 mm × 2.1 mm, 3 μm, Grace, Albany, OR, USA) and the oven temperature was maintained at 30 °C. The eluents were formic acid (0.2%, *v*/*v*; solvent A) and acetonitrile (solvent B). The following gradient was applied: 3% B from 0–5 min, 9% B linear from 5–15 min, 16% B linear from 15–45 min, and 50% B linear from 45–60 min. Then, the column was washed and reconditioned. The flow rate was maintained at 0.2 mL/min, and elution was monitored at 245, 280, 320, and 550 nm for the compounds of interest. The whole effluent (0.2 mL/min) was subsequently injected into the source of the mass spectrometer (MS) without splitting. All MS experiments were carried out in the negative mode. [M − H]-nitrogen was used as nebulizing gas and helium as damping gas. The ion source parameters were as follows: 5.0 kV of spray voltage, 90.0 V of capillary voltage, and 350 °C of temperature. Data were collected and processed using MS Workstation software (V 6.9). The samples were firstly analyzed in full scan mode acquired in the *m*/*z* range 50–2000. MS/MS analyses were performed on a series of selected precursor ions. Finally, the compounds were compared using a database of bioactive compounds (WorkStation version 2.0 database, VARIAN, Palo Alto, CA, USA).

### 3.5. Antioxidant Assays

Antioxidant assays with different reagents were done to determine the potential free radical scavenging activity that polyphenolic compounds obtained from rambutan peel could have. The assays were all carried out in triplicate, and the results were compared with the antioxidant 6-hydroxy-2-5-7-8-Tetramethylchromane-2-Carboxylic acid, commonly known as Trolox (No. 238813, Sigma-Aldrich, Buchs, Switzerland).

The ABTS (2,2′-azino-bis (3-ethylbenzothiazoline-6-sulfonic acid, No. A1888, Sigma-Aldrich, MO, USA) radical scavenging activity of the rambutan peel extract was measured using the method reported by Torres-Leon et al. [51] and Re et al. [52]. The ABTS radical was generated by oxidation of a 7 mM ABTS solution prepared in ethanol, containing 2.45 mM potassium persulfate, then covered adequately in an amber recipient to protect it from light, and stored in the dark for 16 h. Then, the absorbance of the ABTS reagent solution was read at 734 nm (spectrophotometer Biomate 3, Thermo Spectronic, Madison, WI, USA) and adjusted to 0.70 ± 0.02. To measure the scavenging potential, 1 mL of ABTS solution was mixed with 10 μL of the sample in a 1cm-quartz cell. The absorbance was measured at 734 nm after 1 min of reaction time, and a control of ethanol + ABTS was also measured. This was prepared in the same manner as the samples, but ethanol was added instead of the sample. The results were expressed as inhibition percentage:

(1) $$ABTS\;inhibition\;\%=\frac{Ac-As}{Ac}\left(100\right)$$

where *Ac* represents the control absorbance of *ABTS* + ethanol and *As* represents the sample absorbance.

The *DPPH* (2,2-diphenyl-1-picrylhydrazyl) radical scavenging activity was measured using the method of Molyneux [53]. A solution of 60 µM *DPPH* (No. D9132, Sigma-Aldrich, Steinheim, Germany) was diluted in ethanol. Then, 2.9 mL of this *DPPH* solution was mixed with 100 µL of the sample, and the mixture was kept in the dark for 30 min to be able to measure the radical scavenging activity. After that time, the absorbance of the samples with the *DPPH* reagent was measured at 517 nm using a spectrophotometer and a 1-cm quartz cell. The results were then measured as inhibition percentage:

(2) $$DPPH\;inhibition\;\%=\frac{Ac-As}{Ac}\left(100\right)$$

where *Ac* represents the control absorbance of *DPPH* + ethanol and *As* represents the sample absorbance.

The lipid oxidation inhibition (*LOI*) assay was done by using linoleic acid to prove the inhibition activities [54]. The linoleic acid reagent solution was made by mixing 0.56 g of linoleic acid, 1.5 g Tween 20, and 8 mL of 96% ethanol. The samples (50 µL) were mixed with 100 µL of the linoleic acid reagent solution and 1.5 mL of 20 mM acetate buffer at pH 4.0. All samples were incubated at 37 °C, and after 1 min, 750 µL of a 0.5 M FeCl_2_ solution was added to induce oxidation. After 1 h, 1 mL of 0.1 M NaOH solution prepared in 10% *v*/*v* ethanol was added to 250 µL of the previous mixture to stop oxidation. The mixtures were mixed with 2.5 mL of 10% *v*/*v* ethanol and the absorbance was measured in a spectrophotometer at 232 nm with a 1-cm quartz cell. After 24 h of reaction, the same previous process was performed. The results were expressed as lipid oxidation in the samples with the following formula:

(3) $$LOI\left(\%\right)=\frac{\Delta Ac-\Delta As}{\Delta Ac}\left(100\right)$$

where Δ*Ac* is the difference of absorbance between 24 h and 1 h of lipid oxidation in the controls, and Δ*Ac* is the difference of absorbance between 24 h and 1 h lipid oxidation in the samples.

### 3.6. Microbial Inoculum and Prebiotic Assay

Growth stimulation of probiotic bacteria by phenolic compounds present in the extract was evaluated as previously described by Picazo et al. [55]. Bacteria strains were used to evaluate the prebiotic effect of the polyphenolic compounds on *Lactobacillus brevis* and *Lactobacillus paracasei* 25302. Both strains belong to the strain collection of the Food Research Department at the Autonomous University of Coahuila. These bacterial strains were previously activated in MRS media culture and incubated at 37 °C for 24 h. On the day of the experiment, each bacterial culture in exponential growth was diluted in sterile saline solution to a final concentration of 1.5 × 10^8^ CFU/mL. The experiments were performed in MRS broth without glucose and supplemented with rambutan peel polyphenol powder (RPP). Each well was inoculated with 4.17 µL of each bacterial suspension (1.5 × 10^8^ CFU/mL) and RPP fresh serial dilutions (0, 7.8, 15.6, 31.3, 62.5, 125, 250, 500, and 1000 µg/mL) up to 250 µL. Positive (bacteria + medium) and negative (medium) controls, and a blank (PC + medium) were included. The plates were incubated for 24 h at 37 °C, and bacterial growth was then evaluated spectrophotometrically by recording the OD_620nm_ variations and by inoculating into MRS dishes; CFU were determined after incubation for 24 h at 37 °C. All experiments were performed by triplicate. The growth response of each strain on RPP relative to its growth response on glucose was calculated using the method of Kneifel et al. [56]. The calculation was performed using the following formula, where the growth on glucose corresponds to 100%:

(4) $$\mathrm{Relative}\;\mathrm{growth}\;\mathrm{of}\;\mathrm{probiotic}\;\mathrm{strain}\;\mathrm{on}\;\mathrm{RPP}\;\mathrm{substrate}=\left(\frac{A}{B}\right)\times 100\%$$

where *A* is the mean OD_620nm_ of a strain on RPP substrate and *B* is the mean OD_620nm_ of the same strain grown on glucose. In this way, the results reflect the growth of a specific strain on a particular concentration of RPP relative to its growth on a glucose substrate which was fixed at 100%. 

### 3.7. Hemolytic Activity

The hemolysis test was performed according to the method established by Macías-Martínez et al. [57], using fresh human blood from a healthy volunteer donor. The blood was collected in tubes containing EDTA and centrifuged at 2500 rpm for 4 min at 4 °C. The pellet was washed three times with a cold Alsever′s solution (116 mM dextrose, 71 mM sodium chloride, 27 mM sodium citrate, and 2 mM citric acid). The supernatant was diluted in a 1:99 ratio with Alsever′s solution. Then, 150 μL of this suspension were taken for the curve-response experiments. This volume was mixed with rambutan peel extracts (0, 125, 250, 500, and 1000 µg/mL) to evaluate hemolysis. It is noteworthy to mention that the suspension of red blood cells was always freshly prepared and used within 24 h after collection. The tubes were gently mixed in a rotator shaker and then incubated at 36.5 °C in a shaking water bath for 1 h. The Alsever′s solution and deionized water were used as a negative and positive control, respectively. The samples were centrifugated at 3000 rpm for 4 min, and free hemoglobin was measured spectrophotometrically at 415 nm (Sinergy model HTX). The hemolysis percentage was calculated using the following formula:

(5) $$Hemolysis\;\left(\%\right)=\frac{\exp group\;abs-negative\;control}{positive\;control-negative\;control}\;\times \;100$$

where exp *group abs* represent the sample’s absorbance, and positive and negative control represent the absorbance of both the positive and negative controls. The hemolysis assay was performed in triplicate, and the data are representative of two independent experiments.

### 3.8. Statistical Analysis

Statistical analysis was made from the means of three replicates using one-way ANOVA and a Tukey mean comparison test with a significance level of *p* < 0.05 using the software OriginPro 8.5.

## 4. Conclusions

Mexican rambutan peel extract has proven to have not only a potent antioxidant activity, but also a prebiotic effect and non-hemolytic properties. Nowadays, these characteristics are of paramount importance in the development of functional foods and dietary supplements. The importance of these findings relies on the fact that these polyphenolic compounds are being obtained from the peel of the fruit, which is an agricultural waste. The total polyphenolic content of the extract obtained by the hybrid technology (176 mg GAE/g dry rambutan peel) clarifies the gap between using only one technology at a time and using both MAE and UAE, opening the gates for future research not only in the revalorization of rambutan peel, but maybe any other agro-industrial waste. This amount of polyphenolic content provides great antioxidant capacities that surpass even those of Trolox and proves to have prebiotic properties based on the *µMax* value of 0.20 obtained for *paracasei*, a known probiotic bacteria, which was similar to the positive control. With this study, we can begin to demonstrate the importance of revalorizing every single part of the fruit, not only using the pulp, but also producing different products derived from the peel of the fruit, since the polyphenolic compounds obtained have shown antioxidant and prebiotic activity. The evaluation of several characteristics, such as toxicity, proves to be of utmost importance, as applications in food and pharmaceutical fields require rigorous testing. This does not only apply to toxicity, but also the evaluation of other side effects of the polyphenolic content. This study opens the possibility of creating new products in the food, cosmetic, and pharmaceutical industries.

## Figures and Tables

**Table 1:** Identification of obtained compounds from Mexican rambutan′s peel polyphenolic extract.

ID	Retention Time (min)	Compounds	Mass (*m*/*z*)	MS^2^	Group/Family
1	27.37	Dimmers of tergallagic-*O*-hexoside	630.9		Ellagitannin
2	28.67	Corilagin	633.0	481,301,275	Ellagitannin
3	29.50	Geraniin	950.9	933,301,169	Ellagitannin
4	32.73	Ellagic Acid pentoside	433.0	299,300,287,125	Ellagitannin
5	34.81	Ellagic Acid	300.9	257,229,185	Hydroxybenzoic Acid Dimmers

**Table 2:** The antioxidant activity of Mexican rambutan peel extracts expressed as IC_50_, (*) indicates significant differences (*p* < 0.05) between Trolox and Mexican rambutan peel extract. Statistical analysis performed was made to compare the results of the extract against a known antioxidant such as Trolox.

Antioxidant Assay	Trolox (IC_50_ mg/mL)	Mexican Rambutan Peel Extract (IC_50_ mg/mL)
DPPH scavenging effect	0.207 ± 0.001	0.098 ± 0.001 *
ABTS scavenging effect	0.512 ± 0.000	0.335 ± 0.005 *
Lipid oxidation inhibition effect	0.026 ± 0.002	0.034 ± 0.003

**Table 3:** Maximum growth and maximum growth ratio gathered from the prebiotic assay. A Tukey mean comparison test was performed on *L*. *paracasei* and *L*. *brevis* bacterial strains with a significance level of *p* < 0.05 (*), indicating that there is no significant difference between the treatment and positive control.

Bacterial Strain	*L. brevis*	*L. paracasei*
Treatment (ppm) Rambutan Peel Extract	(Maximum Growth) *µMax*	(Maximum Growth) *µMax*
Negative control (custom glucose-free broth)	0.197 ± 0.002	0.189 ± 0.006
7	0.168 ± 0.002	0.135 ± 0.004
15	0.146 ± 0.002	0.185 ± 0.004
31	0.199 ± 0.002	0.190 ± 0.002
62	0.140 ± 0.003	0.187 ± 0.003
125	0.158 ± 0.001	0.195 ± 0.004 *
250	0.127 ± 0.011	0.191 ± 0.004 *
500	0.163 ± 0.005	0.201 ± 0.001 *
1000	0.158 ± 0.001	0.203 ± 0.000 *
Positive control (MRS broth)	0.200 ± 0.001	0.204 ± 0.001

**Table 4:** Hemolytic activity of the polyphenolic extract in isolated human erythrocytes.

Sample (Mexican Rambutan Peel Extract)	Hemolytic Activity [%] ± SD
Negative control (water)	0.032 ± 0.18
125 µg/mL	0.012 ± 0.13
250 µg/mL	0.002 ± 0.20
500 µg/mL	0.016 ± 0.13
1000 µg/mL	0.019 ± 0.04
Positive Control (Alsever)	100 ± 0.08

## Data Availability

The data presented in this study are available in the article.

## References

[1] Castillo-Vera A., Lopez-Guillen G., Sandoval-Esquivez A. (2017). La Historia Del Cultivo de Rambutan (*Nepheliumlapacceum* L.) En México. Agroproductividad.

[2] SAGARPA Boletin de Exportaciones: Rambutan. https://www.gob.mx/cms/uploads/attachment/file/511470/Exportaciones_rambutan_2019.pdf.

[3] Solís-Fuentes J.A., Camey-Ortíz G., del Rosario Hernández-Medel M., Pérez-Mendoza F., Durán-de-Bazúa C. (2010). Composition, Phase Behavior and Thermal Stability of Natural Edible Fat from Rambutan (*Nephelium Lappaceum* L.) Seed. Bioresour. Technol..

[4] Morton J.F. Rambutan. https://hort.purdue.edu/newcrop/morton/rambutan.html.

[5] Hernández-Hernández C., Aguilar C.N., Rodríguez-Herrera R., Flores-Gallegos A.C., Morlett-Chávez J., Govea-Salas M., Ascacio-Valdés J.A. (2019). Rambutan (*Nephelium Lappaceum* L.): Nutritional and Functional Properties. Trends Food Sci. Technol..

[6] Mahmood K., Kamilah H., Alias A.K., Ariffin F. (2018). Nutritional and Therapeutic Potentials of Rambutan Fruit (*Nephelium Lappaceum* L.) and the by-Products: A Review. J. Food Meas. Charact..

[7] Srisawat R., Puengpai S., Nontamart N., Thinkratok A. (2010). Effects of the Crude Extract of the Fruit Rind of Rambutan (*Nephelium Lappaceum* L.) on Blood Pressure, Heart Rate and Respiratory Rate in Anaesthetized Male Rats. Planta Med..

[8] Lestari S.R., Djati M.S., Rudijanto A., Fatchiyah F. (2013). Production and Potency of Local Rambutan at East Java as a Candidate Phytopharmaca. Agrivita.

[9] Kumar S., Chakravart S., Chiew G.S., Subramania T., Palanisamy U., Radhakrish A., Haleagraha N. (2012). Protective Effects of Nephelium Lappaceum Rind Extract against Collagen-Induced Arthritis in Dark Agouti Rats. J. Biol. Sci..

[10] Tadtong S., Athikomkulchai S., Worachanon P., Chalongpol P., Chaichanachaichan P., Sareedenchai V. (2011). Antibacterial Activities of Rambutan Peel Extract. J. Health Res..

[11] Thitilertdecha N., Chaiwut P., Saewan N. (2020). In Vitro Antioxidant Potential of Nephelium Lappaceum, L. Rind Extracts and Geraniin on Human Epidermal Keratinocytes. Biocata Agric. Biotechnol..

[12] Palanisamy U.D., Ling L.T., Manaharan T., Appleton D. (2011). Rapid Isolation of Geraniin from Nephelium Lappaceum Rind Waste and Its Anti-Hyperglycemic Activity. Food Chem..

[13] Thitilertdecha N., Teerawutgulrag A., Kilburn J.D., Rakariyatham N. (2010). Identification of Major Phenolic Compounds from *Nephelium Lappaceum* L. and Their Antioxidant Activities. Molecules.

[14] Perera A., Ton S.H., Palanisamy U.D. (2015). Perspectives on Geraniin, a Multifunctional Natural Bioactive Compound. Trends Food Sci. Technol..

[15] Velázquez-González C., Cariño-Cortés R., Gayosso de Lucio J.A., Ortiz M.I., Arciniega M., Altamirano-Báez D.A., Ángeles L.J., Bautista-Ávila M. (2014). Antinociceptive and Anti-Inflammatory Activities of Geranium Bellum and Its Isolated Compounds. BMC Complement. Altern. Med..

[16] Baliga M.S., Dsouza J.J. (2011). Amla (Emblica Officinalis Gaertn), a Wonder Berry in the Treatment and Prevention of Cancer. Eur. J. Cancer Prev..

[17] Cheng H.S., Ton S.H., Abdul Kadir K. (2017). Ellagitannin Geraniin: A Review of the Natural Sources, Biosynthesis, Pharmacokinetics and Biological Effects. Phytochem. Rev..

[18] Kaderides K., Papaoikonomou L., Serafim M., Goula A.M. (2019). Microwave-Assisted Extraction of Phenolics from Pomegranate Peels: Optimization, Kinetics, and Comparison with Ultrasounds Extraction. Chem. Eng. Process. Intensif..

[19] Mendez-Flores A., Hérnandez-Almanza A., Sáenz-Galindo A., Morlett-Chávez J., Aguilar C.N., Ascacio-Valdés J. (2018). Ultrasound-Assisted Extraction of Antioxidant Polyphenolic Compounds from *Nephelium Lappaceum* L. (Mexican Variety) Husk. Asian Pac. J. Trop. Med..

[20] Simić V.M., Rajković K.M., Stojičević S.S., Veličković D.T., Nikolić N., Lazić M.L., Karabegović I.T. (2016). Optimization of Microwave-Assisted Extraction of Total Polyphenolic Compounds from Chokeberries by Response Surface Methodology and Artificial Neural Network. Sep. Purif. Technol..

[21] Jovanovic A., Petrovic P., Ðordjevic V., Zdunic G., Savikin K., Bugarski B. (2017). Polyphenols Extraction from Plant Sources. Lek. Sirovine.

[22] Martina K., Tagliapietra S., Barge A., Cravotto G. (2016). Combined Microwaves/Ultrasound, a Hybrid Technology. Top. Curr. Chem..

[23] Ordoñez-Torres A., Torres-León C., Hernández-Almanza A., Flores-Guía T., Luque-Contreras D., Aguilar C.N., Ascacio-Valdés J. (2021). Ultrasound-Microwave-Assisted Extraction of Polyphenolic Compounds from Mexican “Ataulfo” Mango Peels: Antioxidant Potential and Identification by HPLC/ESI/MS. Phytochem. Anal..

[24] Hernández-Hernández C., Aguilar C.N., Flores-Gallegos A.C., Sepúlveda L., Rodríguez-Herrera R., Morlett-Chávez J., Govea-Salas M., Ascacio-Valdés J. (2020). Preliminary Testing of Ultrasound/Microwave-Assisted Extraction (U/M-AE) for the Isolation of Geraniin from *Nephelium Lappaceum* L. (Mexican Variety) Peel. Processes.

[25] Khadhraoui B., Ummat V., Tiwari B.K., Fabiano-Tixier A.S., Chemat F. (2021). Review of Ultrasound Combinations with Hybrid and Innovative Techniques for Extraction and Processing of Food and Natural Products. Ultrason. Sonochem..

[26] Hernández C., Ascacio-Valdés J., De la Garza H., Wong-Paz J., Aguilar C.N., Martínez-Ávila G.C., Castro-López C., Aguilera-Carbó A. (2017). Polyphenolic Content, in Vitro Antioxidant Activity and Chemical Composition of Extract from *Nephelium Lappaceum* L. (Mexican Rambutan) Husk. Asian Pac. J. Trop. Med..

[27] Phuong N.N.M., Le T.T., Dang M.Q., Van Camp J., Raes K. (2020). Selection of Extraction Conditions of Phenolic Compounds from Rambutan (*Nephelium Lappaceum* L.) Peel. Food Bioprod. Process..

[28] Li X., Deng Y., Zheng Z., Huang W., Chen L., Tong Q., Ming Y. (2018). Corilagin, a Promising Medicinal Herbal Agent. Biomed. Pharmacother..

[29] Attar R., Birsu Cincin Z., Sinem Bireller E., Cakmakoglu B. (2017). Apoptotic and Genomic Effects of Corilagin on SKOV3 Ovarian Cancer Cell Line. Onco Targets Ther..

[30] Liu F.-C., Chaudry I.H., Yu H.-P. (2017). Hepatoprotective effects of corilagin following hemorrhagic shock are through akt-dependent pathway. Shock.

[31] Yoganathan S., Alagaratnam A., Acharekar N., Kong J. (2021). Ellagic Acid and Schisandrins: Natural Biaryl Polyphenols with Therapeutic Potential to Overcome Multidrug Resistance in Cancer. Cells.

[32] Ríos J.L., Giner R.M., Marín M., Recio M.C. (2018). A Pharmacological Update of Ellagic Acid. Planta Med..

[33] Yang Y., Zhang L., Fan X., Qin C., Liu J. (2012). Antiviral Effect of Geraniin on Human Enterovirus 71 in Vitro and in Vivo. Bioorg. Med. Chem. Lett..

[34] Londhe J.S., Devasagayam T.P.A., Foo L.Y., Shastry P., Ghaskadbi S.S. (2012). Geraniin and Amariin, Ellagitannins from Phyllanthus Amarus, Protect Liver Cells against Ethanol Induced Cytotoxicity. Fitoterapia.

[35] Li J., Wang S., Yin J., Pan L. (2013). Geraniin Induces Apoptotic Cell Death in Human Lung Adenocarcinoma A549 Cells in Vitro and in Vivo. Can. J. Physiol. Pharmacol..

[36] Ling L.T., Radhakrishnan A.K., Subramaniam T., Cheng H.M., Palanisamy U.D. (2010). Assessment of Antioxidant Capacity and Cytotoxicity of Selected Malaysian Plants. Molecules.

[37] Parkar S.G., Stevenson D.E., Skinner M.A. (2008). The Potential Influence of Fruit Polyphenols on Colonic Microflora and Human Gut Health. Int. J. Food Microbiol..

[38] Jiao X., Wang Y., Lin Y., Lang Y., Li E., Zhang X., Zhang Q., Feng Y., Meng X., Li B. (2019). Blueberry Polyphenols Extract as a Potential Prebiotic with Anti-Obesity Effects on C57BL/6 J Mice by Modulating the Gut Microbiota. J. Nutr. Biochem..

[39] García-Ruiz A., Bartolomé B., Martínez-Rodríguez A.J., Pueyo E., Martín-Álvarez P.J., Moreno-Arribas M.V. (2008). Potential of Phenolic Compounds for Controlling Lactic Acid Bacteria Growth in Wine. Food Control..

[40] Jiménez N., Curiel J.A., Reverón I., de las Rivas B., Muñoz R. (2013). Uncovering the Lactobacillus Plantarum WCFS1 Gallate Decarboxylase Involved in Tannin Degradation. Appl. Environ. Microbiol..

[41] Bhat T.K., Singh B., Sharma O.P. (1998). Microbial Degradation of Tannins—A Current Perspective. Biodegradation.

[42] Selma M.V., Beltrán D., García-Villalba R., Espín J.C., Tomás-Barberán F.A. (2014). Description of Urolithin Production Capacity from Ellagic Acid of Two Human Intestinal Gordonibacter Species. Food Funct..

[43] Cyboran-Mikołajczyk S., Csonka Á., Molnar J., Szabó D., Oszmiański J., Kleszczyńska H. (2018). In Vitro Studies of Anti-Hemolytic and Cytotoxic Activity of Procyanidin-Rich Extract from the Leaves of Actinidia Arguta. Polish J. Food Nutr. Sci..

[44] Ajay Krishna P.G., Sivakumar T.R., Jin C., Li S.-H., Weng Y.-J., Yin J., Jia J.-Q., Wang C.-Y., Gui Z.Z. (2018). Antioxidant and Hemolysis Protective Effects of Polyphenol-Rich Extract from Mulberry Fruits. Pharmacogn. Mag..

[45] Halla N., Boucherit K., Zeragui B., Djelti A., Belkhedim Z., Hassani R., Benatallah S., Djellouli H., Kacimi O., Boucherit-Otmani Z. (2020). Polyphenols Content and Antimicrobial, Antioxidant and Hemolytic Activities of Essential Oils from Four Selected Medicinal Plants Growing in Algeria. Biol. Med. Nat. Prod. Chem..

[46] Olchowik E., Lotkowski K., Mavlyanov S., Abdullajanova N., Ionov M., Bryszewska M., Zamaraeva M. (2012). Stabilization of Erythrocytes against Oxidative and Hypotonic Stress by Tannins Isolated from Sumac Leaves (*Rhus Typhina* L.) and Grape Seeds (*Vitis Vinifera* L.). Cell. Mol. Biol. Lett..

[47] Routray W., Orsat V. (2012). Microwave-Assisted Extraction of Flavonoids: A Review. Food Bioprocess. Technol..

[48] Musci M., Yao S. (2017). Optimization and Validation of Folin–Ciocalteu Method for the Determination of Total Polyphenol Content of Pu-Erh Tea. Int. J. Food Sci. Nutr..

[49] Shay P.E., Trofymow J.A., Constabel C.P. (2017). An Improved Butanol-HCl Assay for Quantification of Water-Soluble, Acetone: Methanol-Soluble, and Insoluble Proanthocyanidins (Condensed Tannins). Plant. Methods.

[50] Ascacio-Valdés J.A., Aguilera-Carbó A.F., Buenrostro J.J., Prado-Barragán A., Rodríguez-Herrera R., Aguilar C.N. (2016). The Complete Biodegradation Pathway of Ellagitannins by Aspergillus Niger in Solid-State Fermentation. J. Basic Microbiol..

[51] Torres-León C., Ramírez-Guzmán N., Ascacio-Valdés J., Serna-Cock L., dos Santos Correia M.T., Contreras-Esquivel J.C., Aguilar C.N. (2019). Solid-State Fermentation with Aspergillus Niger to Enhance the Phenolic Contents and Antioxidative Activity of Mexican Mango Seed: A Promising Source of Natural Antioxidants. LWT.

[52] Re R., Pellegrini N., Proteggente A., Pannala A., Yang M., Rice-Evans C. (1999). Antioxidant activity applying an improved abts radical cation decolorization assay. EMPA Act..

[53] Molyneux P. (2004). The Use of the Stable Free Radical Diphenylpicryl-Hydrazyl (DPPH) for Estimating Antioxidant Activity. Songklanakarin J. Sci. Technol..

[54] Martínez-Ávila G.C., Aguilera-Carbó A.F., Rodríguez-Herrera R., Aguilar C.N. (2012). Fungal Enhancement of the Antioxidant Properties of Grape Waste. Ann. Microbiol..

[55] Picazo B., Flores-Gallegos A.C., Ilina A., Rodríguez-Jasso R.M., Aguilar C.N. (2019). Production of an Enzymatic Extract from Aspergillus Oryzae Dia-Mf to Improve the Fructooligosaccharides Profile of Aguamiel. Front. Nutr..

[56] Kneifel W. (2000). In Vitro Growth Behaviour of Probiotic Bacteria in Culture Media with Carbohydrates of Prebiotic Importance. Microb. Ecol. Health Dis..

[57] Macías-Martínez B.I., Cortés-Hernández D.A., Zugasti-Cruz A., Cruz-Ortíz B.R., Múzquiz-Ramos E.M. (2016). Heating Ability and Hemolysis Test of Magnetite Nanoparticles Obtained by a Simple Co-Precipitation Method. J. Appl. Res. Technol..

